# Implementing apportionment strategy to identify costs in a multidisciplinary clinic

**DOI:** 10.1590/S1679-45082017GS3759

**Published:** 2017

**Authors:** Renato Ribeiro Nogueira Ferraz, Anna Sofia Costa Neri, Estela Capelas Barbosa, Marcus Vinícius Cesso da Silva

**Affiliations:** 1 Universidade Nove de Julho, São Paulo, SP, Brazil.

**Keywords:** Health Management, Costs and cost analysis, Cost allocation, Ambulatory care facilities/economics, Strategies

## Abstract

**Objective:**

To present the implementation of an apportionment strategy proportional to the productive areas of a multidisciplinary clinic, defining the minimum values to be passed monthly to health professionals who work there.

**Methods:**

A study of the clinic structure was carried out, in which the area of occupation of each service was defined. Later the cost was prorated, allocating a value to each room, proportional to the space occupied.

**Results:**

The apportionment implementation allowed the clinic managers to visualize the cost of each room, providing a value base for formation of a minimum amount necessary to be passed monthly to each professional, as a form of payment for rent of using their facilities.

**Conclusion:**

The risk of financial loss of the clinic was minimized due to variation of its productivity, as well as the conditions of transference at the time of hiring by professionals were clear, promoting greater confidence and safety in contract relations.

## RESUMO

**Objetivo:**

Apresentar a implantação de uma estratégia de rateio proporcional às áreas produtivas de uma clínica multidisciplinar, definindo valores mínimos a serem repassados mensalmente aos profissionais de saúde que as ocupam.

**Métodos:**

Estudo da estrutura da clínica, no qual foi definida, em metros quadrados, a área de ocupação de cada serviço. Em seguida, o custo foi rateado, alocando um valor a cada sala, proporcional ao espaço ocupado.

**Resultados:**

A implantação do rateio possibilitou aos gestores da clínica estudada visualizar o custo de cada sala, fornecendo uma base de valor para formação de um valor mínimo necessário a ser repassado mensalmente para cada profissional, como forma de pagamento pelo aluguel de utilização de suas instalações.

**Conclusão:**

Minimizou-se o risco de prejuízo da clínica pela variação de sua produtividade, bem como ficaram claras as condições de repasse no momento de contratação do aluguel pelos profissionais, promovendo maior confiança e segurança na relação contratual.

## INTRODUCTION

Over the last 30 years, the field of administration has dwelt on the development and perfecting of the theory of costs. At the start of the 20th century, Taylor and Ford were insightful when they pointed out the importance of increased productivity of workers and reduction of pores of work.^1^ Initially, professional administrators focused on increased productivity via technological innovation, deriving from this the rapid development of cybernetics and computer sciences.^2^ However, with the oil crisis and restructuring of the production chain during the 1970’s and 1980’s,^3^ the need to increase the worker productivity by reducing costs became evident.

Cost is understood as a “expense related to goods or services used in the production of other goods and services”.^4^ For Nascimento,^5^ “cost is the sum of goods and services consumed or used in the production of new goods or services, translated into monetary units”. According to Abbas,^6^ “cost represents the value of goods and services consumed in the production of other goods or services”. First, it was the industries that went through the process of cost determination, followed by agribusiness and finally, by the services industry. Even though the large organizations have currently determined the direct and indirect costs of their work processes, in family business this is still not the rule, and it has expanded little by little.

The clinic, which is the object of study of this report, is located in the city of Arapiraca (AL), known as the main city of the interior of the State. On 2016 it had approximately 220 thousand inhabitants and was known as an important commercial center of the rustic region, where the second most significant metropolitan region of the State was located. This was composed of 20 cities, and Arapiraca was a reference city, directly influencing a population of approximately 500 thousand inhabitants.

The clinic was in the center of the city, a location easily accessible to public transportation and with a concentration of commercial establishments. Its structure was made up of various services, from medical offices to audiometric testing rooms, laboratory specimen collections, procedures, physical therapy, and ultrasound.

The clinic was created with the intention of meeting the demand of patients who had no health insurance or financial means to pay for the private procedures, although they had the financial means to cover lower prices, and would even pay immediately. This type of business has proven very competitive in the region and has piqued the interest of healthcare professionals, who require a comfortable and safe environment in which to care for their patients, with no need to set up a private practice. For this, they make partnerships with clinics that have this profile to deliver their services.

Since its foundation, the managers established as form of payment for the use of the clinic structure a percentage of the value of care given by the professional. This value was used by the clinic managers to pay expenses for running the structure. However, as the clinic initiated its activities, it was evident that the value transferred coming from the appointments was not sufficient to cover the costs of the clinic; moreover, they varied a lot, since it depended directly on the volume of patients seen by each professional.

In healthcare, payment according to production is not uncommon − even the Unified Health System (SUS – *Sistema* Único *de Saúde*) did that soon after its implantation.^7^ This payment system presents a clear disadvantage of producing moral risk and reducing the incentive for the business owners, since the production is not directly under their control. In the case of this clinic, the return visits to the clinic could imply moral risk, since they are not charged, which gives the patient an incentive to return more often than necessary, thus increasing the costs of the clinic.

With the intention of correcting the problem, the implantation of an apportionment model was adopted, considering the square meters occupied by each service. The clinic was divided into areas denominated productive, and the others were called support areas. The total costs of the clinic during the period of one semester were surveyed, and based on these values, a monthly average cost for the clinic was reached, which was used as an initial value for negotiation with the professionals that rent the rooms, after the division of the value prorated to the area used by the diverse services provided at the clinic.

## OBJECTIVE

To present the implantation of a strategy of apportionment prorated to the productive areas of a multidisciplinary clinic, defining minimum values to be transferred monthly to the healthcare professionals that occupy them.

## METHODS

The research strategies used for carrying out this project were bibliographic search, with literature review for theoretical foundation of the concepts; and action-research, defined as “a type of initial investigation with the peculiar characteristic of having the purpose of the action planned around the problems detected”.^8^

When evaluating the financial return the professionals would transfer per volume of cases seen in payment of the structure, the managers perceived the values did not match the costs of the clinic. Another form of defining a value for each space was needed in order to guarantee to the clinic the receipt of a minimum value of payment for its expenses, eliminating the possibility of financial losses.

Thus, a survey of the costs and expenses of the clinic was made with the objective of composing the total cost, and identifying the value to be prorated among its sectors. Costs and expenses, both fixed and variable, direct and indirect were taken into consideration. Since there is a small variation in the value of the total cost on different months, the value of this cost was determined using as basis the mean of the costs of the previous semester, that is, the estimated cost, defined as “fixed based on values used during the previous period and established quantitative production and sales”.^5^

The production cost of the service was determined by the method of cost by absorption, that imputes to the final product, or to the production, all the direct variable costs added to the indirect and fixed costs.^5^ Given the intangible nature of the services, the method of total absorption cost seemed most appropriate.^9^ The method of cost allocation used was the direct method, which is characterized by allocation of costs directly to the operational services, ignoring the services rendered by one department to the other.^10^ The use of square meter as unit for cost allocation in apportionments is often adopted as a criterion for the apportionment of the value of rent among the occupants of a building, and the apportionment per hour used to allocate the costs of man labor.^5^

In this composition, the costs of infrastructure and fixed expenses, such as the value of rent for the building, contracts of outsourced security and information technology services, among others, were considered to compose the allotment by square meter. The variable costs and expenses, such as water, electricity, and telephone bills, and expenses of office materials and cleaning products, among others, comprised the apportionment per hour of occupation. Still considering the variable costs, the expenses with personnel were included, which should be prorated per hour of use of the structure.^4,11^ The depreciation values of the medical equipment were allocated only to the rooms in which they were located. Based on a six-month history of total costs of the clinic, the average costs were calculated, aiming to identify a mean cost that served as basis for the apportionment. We point out that his project did not detail the bills that made up these costs, since the primary objective was to present the apportionment.

After adding up the total cost, an analysis was made of the physical structure of the clinic, which was divided into support areas and productive areas. The support areas comprised two waiting rooms and two restrooms for clients; an area for the employees, including dressing rooms, restrooms, and kitchen; administrative area with storage room and administration room; an area to store cleaning materials and waste disposal. The following areas were defined as productive: eight medical offices; ultrasound room; physical therapy room; audiometric testing room; laboratory sample collection room; and procedure room (used for dressings and minor surgical procedures). Among the eight medical offices, one was specific for the psychology team.

After evaluation of the physical structure, the physical space occupied by each sector was evaluated by means of a study of the floor plan of the building. The measurement of each productive room was made in square meters. The next step, by means of the direct method of cost allocation, the value of each room was calculated, based on the total cost of the clinic, identified previously. The support area was not considered for cost allocation, since these were common areas, used by the clients and employees of the clinic in general, and should be absorbed by the productive sectors since they were a part of the structure composed to provide care. Thus, a spreadsheet was prepared presenting the total area of the clinic and its division among the other areas, as well as its percentage relative to the total area. Based on this division, the total cost of the clinic was prorated, attributing to each sector the value of the room, considering the percentage area occupied at the clinic.

## RESULTS

The evaluation of the floor plan of the building and the division of areas per square meter showed the support area occupied 204.46m^2^, accounting for 62% of total area of the clinic physical structure. The remaining 123.06m^2^ made up the rest of the clinic area, accounting for 48% of the area considered productive, on which the apportionment was applied (the support area was understood as part of the structure rented for rendering care).

In dividing the areas, it was noted that most rooms had an average area of 10m^2^ − or 3% when considering the total area of the clinic. Considering that only the productive areas were relevant to calculate the apportionment, its proportional relevance went to 9% of area. The smaller areas were allocated in the psychology office and in the procedure room.

It is interesting to note the importance of the support areas in the structure of the clinic − in this case, 62% of the total built area (Table 1). Such a fact does not occur only in multidisciplinary clinics and may be extrapolated to many segments of the services industry. Services are basically characterized by no division between consumption and production, since both occur simultaneously. However, for this to be possible, it is necessary to have a physical structure that allows the execution of the service, as this is the primary function of the support area.

**Table 1:** Apportionment per square meter of productive area in a multidisciplinary clinic

Sectors	Built area	Productive area	Cost of the area	Cost of the area/	Cost of the area/	Cost of the area/	Cost of the area/	Cost of the area/
	(%)	(%)	(R$)	Month (U$)*	Day (R$)	Day (U$)	Hour (R$)	Hour (U$)
Medical office 1	12.13 (4)	12.13 (10)	981,76	302.08	49,09	15.10	4,09	1.26
Medical office 2	10.55 (3)	10.55 (9)	853,88	262.73	42,69	13.14	3,56	1.10
Medical office 3	10.51 (3)	10.51 (9)	850,64	261.74	42,53	13.09	3,54	1.09
Medical office 4	8.55 (3)	8.55 (7)	692,00	212.92	34,60	10.65	2,88	0.89
Medical office 5	8.39 (3)	8.39 (7)	679,05	208.94	33,95	10.45	2,83	0.87
Psychology office	7.12 (2)	7.12 (6)	576,27	177.31	28,81	8.86	2,40	0.74
Audiometrics office	10.33 (3)	10.33 (8)	836,07	257.25	41,80	12.86	3,48	1.07
Medical office 6	17.18 (5)	17.18 (14)	1.390,48	427.84	69,52	21.39	5,79	1.78
Procedure room	6.01 (2)	6.01 (5)	486,43	149.67	24,32	7.48	2,03	0.62
Ultrasound room	10.33 (3)	10.33 (8)	836,07	257.25	41,80	12.86	3,48	1.07
Laboratory	10.8 (3)	10.8 (9)	874,11	268.96	43,71	13.45	3,64	1.12
Physical therapy	11.16 (3)	11.16 (9)	903,25	277.92	45,16	13.90	3,76	1.16
Productive support area	204.46 (62)							

Total	327.52 (100)	123.06 (100)	9.960,00	3,064.62	498,00	153.22	41,50	12.76

* The American dollar was considered at the exchange rate of R$ 3,25 (July 2016).

In the presentation of costs prorated hour of production, it was noted that the total costs of services (R$ 22.653,33) represented an amount 127% superior to the total costs prorated by square meter (R$ 9.960,00) (Table 2).

**Table 2:** Apportionment per hour of production

Sectors	Productive area	Cost of services/	Cost of services/	Cost of services/	Cost of services/	Cost of services/	Cost of services/
	(%)	Month (R$)	Month (U$)*	Day (R$)	Day (U$)	Hour (R$)	Hour (U$)
Medical office 1	12.13 (10)	2.232,93	687.06	111,65	34.35	9,30	2.86
Medical office 2	10.55 (9)	1.942,08	597.56	97,10	29.88	8,09	2.49
Medical office 3	10.51 (9)	1.934,72	595.30	96,74	29.77	8,06	2.48
Medical office 4	8.55 (7)	1.573,91	484.28	78,70	24.22	6,56	2.02
Medical office 5	8.39 (7)	1.544,46	475.22	77,22	23.76	6,44	1.98
Psychology office	7.12 (6)	1.310,68	403.29	65,53	20.16	5,46	1.68
Audiometrics office	10.33 (8)	1.901,58	585.10	95,08	29.26	7,92	2.44
Medical office 6	17.18 (14)	3.162,56	973.10	158,13	48.66	13,18	4.06
Procedure room	6.01 (5)	1.106,34	340.41	55,32	17.02	4,61	1.42
Ultrasound room	10.33 (8)	1.901,58	585.10	95,08	29.26	7,92	2.44
Laboratory	10.8 (9)	1.988,10	611.72	99,41	30.59	8,28	2.55
Physical therapy	11.16 (9)	2.054,37	632.11	102,72	31.61	8,56	2.63
Productive support area							

Total	123.06	22.653,33	6,970.25	1.132,67	348.52	94,39	29.04

* The American dollar was considered at an exchange rate of R$ 3,25 (July 2016).

When we gear our attention towards the apportionment of the indirect costs per productive area, we note a minimum value of R$ 1.106,34 and a maximum value of R$ 3.162,56 (Table 2).

The cost of the rooms varied between R$ 2.300,00 and R$ 4.600,00 per month (Table 3). When detailing this calculation even further, we found the values per day and per hour, easing the calculations for the presentation of values to the professionals interested in renting the spaces to render their services, since they were already used to renting rooms per hour of use.

**Table 3:** Room cost per hour of use

Sectors	Area in meters	Cost of room/	Cost of room/	Cost of room/	Cost of room/	Cost of room/	Cost of room/
	(%)	Month (R$)	Month (U$)*	Day (R$)	Day (U$)	Hour (R$)	Hour (U$)
Medical office 1	12.13 (10)	3.214,69	989.14	160,73	49.46	13,39	4.12
Medical office 2	10.55 (9)	2.795,96	860.30	139,80	43.02	11,65	3.58
Medical office 3	10.51 (9)	2.785,36	857.03	139,27	42.85	11,61	3.57
Medical office 4	8.55 (7)	2.265,92	697.21	113,30	34.86	9,44	2.90
Medical office 5	8.39 (7)	2.223,52	684.16	111,18	34.21	9,26	2.85
Psychology office	7.12 (6)	1.886,94	580.60	94,35	29.03	7,86	2.42
Audiometrics office	10.33 (8)	2.737,65	842.35	136,88	42.12	11,41	3.51
Medical office 6	17.18 (14)	4.553,04	1,400.94	227,65	70.05	18,97	5.84
Procedure room	6.01 (5)	1.592,77	490.08	79,64	24.50	6,64	2.04
Ultrasound room	10.33 (8)	2.737,65	842.35	136,88	42.12	11,41	3.51
Laboratory	10.8 (9)	2.862,21	880.68	143,11	44.03	11,93	3.67
Physical therapy	11.16 (9)	2.957,62	910.04	147,88	45.50	12,32	3.79
Productive support area							

Total	123.06	32.613,33	10,034.87	1.630,67	501,74	135,89	41.81

* The American dollar was considered at an exchang rate of R$ 3,25 (July 2016).

## DISCUSSION

In the field of health, cost analysis is complex due to the diversity of types of patient, care given, management or even the treatment to be adopted for each patient, which varies with age, sex, and basic health status.^12^

There was the idea that the professionals, when working by production, would be interested in seeing many patients, increasing their gains and consequently, transfer the percentage destined to the clinic. This, however, was not confirmed at the clinic under study because the professionals worked at several locations. Moreover, since there was no minimum value to be transferred, there was no concern on the part of the professionals when they were not needed at the clinic, for they would not have to transfer to it the value relative to the period in which the room they used was empty, and thus were able to dedicate themselves to other activities.

Among the results found in this study, the great difference in value between the costs prorated per hour of production and the costs per square meters can be attributed to the fact that the cost of payroll is the superior value, since it accounted for 46% of total clinic cost. This relation with the high value of payment to the professionals may be seen in an investigation comparing costs between two modalities of healthcare units for newborns, conducted by Entringer et al.,^13^who demonstrated the payroll accounting for 77-78% of the costs identified for services rendered.

In the apportionment of the indirect costs per productive area, the greater value was practically double the mean cost; and it was only justified by the administrative decision of the owner of the clinic to keep a larger medical office for him, despite the encumbrance that this might cause.

The values found in this study allowed the clinic managers to identify if the values calculated as percentage to be transferred for each procedure performed to payment of rent of the rooms was sufficient to pay the costs of the clinic, and thus, maintain its survival and longevity. Nevertheless, the rooms need to have a satisfactory level of occupation to generate a volume of cases seen sufficient to pay for the costs, or alternately, the professional should be responsible for the cost of renting the space, even when the transfer is smaller.

Therefore, this information gave the managers greater clarity and arguments at the time of defining and presenting the financial duties of the professionals, since regardless of their production, the contracting professional is aware of a minimal amount to be paid monthly. On the other hand, the clinic assures receiving a value that allows it to remain active and with no losses stemming from variability in patient load of the contracting healthcare professional.

## CONCLUSION

The implementation of the apportionment presented in this article enabled the managers of a multidisciplinary clinic to visualize the cost of each room, providing the basis for a minimum value necessary to be transferred monthly by each professional, as a form of payment for rent of facilities.
